# Sonic hedgehog lineage in the mouse hypothalamus: from progenitor domains to hypothalamic regions

**DOI:** 10.1186/1749-8104-7-4

**Published:** 2012-01-20

**Authors:** Gonzalo Alvarez-Bolado, Fabian A Paul, Sandra Blaess

**Affiliations:** 1 Department of Neuroanatomy, University of Heidelberg, Im Neuenheimer Feld 307, 69120 Heidelberg, Germany; 2 Institute of Reconstructive Neurobiology, Life and Brain Center, University of Bonn, Sigmund-Freud-Str. 25, 53127 Bonn, Germany; 3 Max-Delbrück-Centrum for Molecular Medicine (MDC), Robert-Rössle-Str. 10, 13092 Berlin, Germany

## Abstract

**Background:**

The hypothalamus is a brain region with essential functions for homeostasis and energy metabolism, and alterations of its development can contribute to pathological conditions in the adult, like hypertension, diabetes or obesity. However, due to the anatomical complexity of the hypothalamus, its development is not well understood. *Sonic hedgehog *(*Shh*) is a key developmental regulator gene expressed in a dynamic pattern in hypothalamic progenitor cells. To obtain insight into hypothalamic organization, we used genetic inducible fate mapping (GIFM) to map the lineages derived from *Shh-*expressing progenitor domains onto the four rostrocaudally arranged hypothalamic regions: preoptic, anterior, tuberal and mammillary.

**Results:**

*Shh-*expressing progenitors labeled at an early stage (before embryonic day (E)9.5) contribute neurons and astrocytes to a large caudal area including the mammillary and posterior tuberal regions as well as tanycytes (specialized median eminence glia). Progenitors labeled at later stages (after E9.5) give rise to neurons and astrocytes of the entire tuberal region and in particular the ventromedial nucleus, but not to cells in the mammillary region and median eminence. At this stage, an additional *Shh*-expressing domain appears in the preoptic area and contributes mostly astrocytes to the hypothalamus. *Shh-*expressing progenitors do not contribute to the anterior region at any stage. Finally, we show a gradual shift from neurogenesis to gliogenesis, so that progenitors expressing Shh after E12.5 generate almost exclusively hypothalamic astrocytes.

**Conclusions:**

We define a fate map of the hypothalamus, based on the dynamic expression of *Shh *in the hypothalamic progenitor zones. We provide evidence that the large neurogenic *Shh-*expressing progenitor domains of the ventral diencephalon are continuous with those of the midbrain. We demonstrate that the four classical transverse zones of the hypothalamus have clearly defined progenitor domains and that there is little or no cell mixing between the tuberal and anterior or the preoptic and anterior hypothalamus. Finally, we show that, in the tuberal hypothalamus, neurons destined for every mediolateral level are produced during a period of days, in conflict with the current 'three-wave' model of hypothalamic neurogenesis. Our work sets the stage for a deeper developmental analysis of this complex and important brain region.

## Background

The hypothalamus is essential for the maintenance of homeostasis and the survival of the individual and the species because of its central role in the regulation of eating, drinking, reproductive and parental behavior, as well as sleep-wake rhythms. It fulfills these functions by controlling the autonomic nervous system and hormone secretion; in addition, the hypothalamus integrates input from all other brain regions and the peripheral nervous system [1,2]. The hypothalamus consists of a wide variety of neuronal clusters with distinct connectivity and function, classically subdivided along the rostral-caudal axis into four regions - preoptic, anterior, tuberal and mammillary - and along the medial-lateral axis into periventricular, medial and lateral zones [3].

The hypothalamus develops from the rostral diencephalon after induction by the underlying prechordal plate. The secreted morphogen Sonic hedgehog (Shh), initially expressed in the prechordal plate, is essential for this inductive process. *Shh *null mutant mice lack all hypothalamic structures [4,5] and Hedgehog protein can induce hypothalamic tissue in forebrain explants [6,7]. After induction, the presumptive hypothalamus is patterned along the three spatial axes in order to define distinct hypothalamic areas. Shh, which is expressed in a dynamic pattern in the developing ventral hypothalamus, plays a role in these patterning events as well [8,9]. A recent study in which *Shh *was specifically inactivated in the neuroepithelium, but not in the prechordal plate, demonstrated that neural Shh plays a role in ventrodorsal and rostrocaudal patterning and in establishing lateral hypothalamic neurons but does not seem to be required for regional hypothalamic specification and eyefield separation, for which non-neural Shh signaling from the prechordal plate appears to be sufficient [10].

Although numerous studies explore the functional and anatomical complexity of the hypothalamus, relatively little is known about how the diverse array of hypothalamic cell types is generated and organized during development. Even though some of the molecules regulating hypothalamic development have been identified, it remains unclear how these signals are integrated to direct the specification of the large number of distinct hypothalamic cell types. Moreover, the relationship between precursor domains defined by the expression of specific markers and the cell types in the mature hypothalamus remains largely undefined [11], except for those neuroepithelial regions expressing Pou3 transcription factors [12]. Genetic defects in the induction and specification of distinct hypothalamic cell types might result in metabolic or homeostatic disorders and there is evidence that this is the case in congenital obesity [13-15]. Identifying the developmental origin and lineage relationships of hypothalamic neurons is the first and essential step in shedding light on common pathological processes such as hypertension, diabetes and obesity.

Here, in order to establish a detailed fate map of the hypothalamus, we analyzed the fate of cells derived from *Shh-*expressing hypothalamic progenitors using genetic inducible fate mapping (GIFM) to permanently mark cells at different embryonic stages (embryonic day (E)7.5 to E12.5) [16]. We show that, before E9.5, *Shh-*expressing progenitors are primarily located in the ventral midline of the posterior hypothalamic primordium and give rise to neurons and astrocytes throughout the mammillary and posterior tuberal regions, but also to tanycytes, a specialized glial cell type in the median eminence of the tuberal region. After E9.5, *Shh-*expressing progenitors are still found in the posterior hypothalamic anlage, but become progressively rarer in the most posterior area and ventral midline. This lateral progenitor domain only contributes few cells to the mammillary region and median eminence. However, this domain gives rise to neurons and astrocytes of the tuberal region, in particular the ventromedial nucleus, indicating that the tuberal hypothalamus is largely derived from lateral progenitors. In addition, a progenitor domain appears in the presumptive preoptic region whose contribution to the hypothalamus consists mostly of preoptic astrocytes. At any labeling stage, we observed very few *Shh-*expressing progenitors in the area between the posterior hypothalamic primordium and the presumptive preoptic region and accordingly only few fate-mapped cells in the anterior hypothalamus in the adult brain. At late labeling time points there is a shift from neurogenesis to gliogenesis, so that by E12.5, *Shh-*expressing progenitors give rise almost exclusively to hypothalamic astrocytes. Finally, cells derived from progenitors that express Shh at late stages (E10.5 and E11.5) contribute neurons to all mediolateral zones of the tuberal hypothalamus, indicating that the current 'three-wave model', according to which late-born hypothalamic neurons remain in the medial hypothalamus, might have to be revised [17].

## Results

### Sonic hedgehog expression in the ventral diencephalon is dynamic

**Figure 1:**
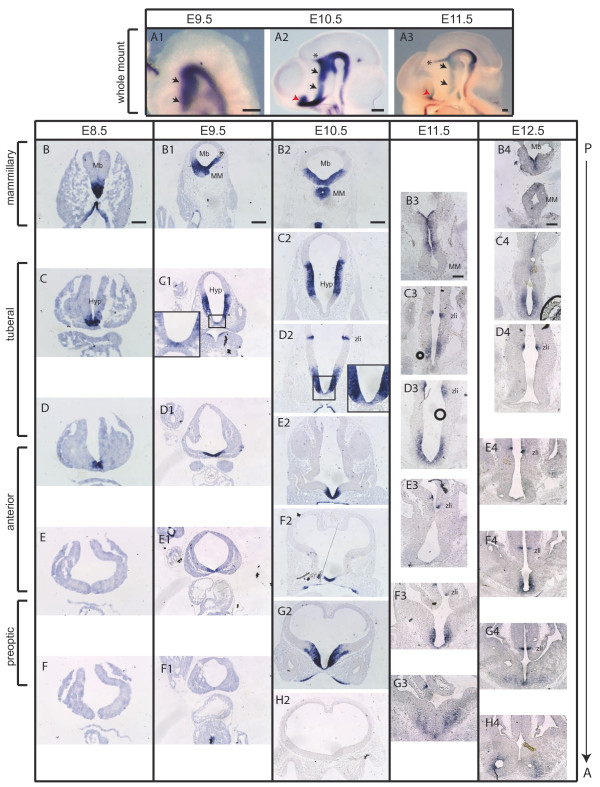
**The dynamic expression pattern of Shh in the hypothalamic primordium**. **(A-H) **RNA *in situ *hybridization in whole mounts (A_1_-A_3_) or on transverse sections of the hypothalamic primordium (hyp) at several anterior-posterior levels (B-H_4_). *Shh *is initially expressed in the ventral midline in the posterior hyp at E8.5 (black arrows in A_1_-A_3_; B-F). *Shh *expression expands laterally over the subsequent days of development and starts to be downregulated in the ventral midline (E9.5 to E12.5) (A_1_-H_4_). At E9.5 to E12.5 Shh is also expressed in the preoptic area and the zona limitans intrathalamica (zli). Red arrowheads in (A_2_, A_3_) indicate the preoptic area, and asterisks indicate the zli. Insets in (C_1_, D_2_): higher magnifications of the boxed area. Mb, midbrain; MM, mammillary primordium. Scale bars: 80 μm (B-F); 200 μm (A_1_-A_3_, B_1_-H_4_).

It has previously been shown that Shh expression is dynamic in the hypothalamus [10,18], and that these spatial-temporal expression changes are of great functional importance [8,9]. To gain a detailed insight into the temporally dynamic expression pattern of *Shh *in the developing murine hypothalamus, we performed *in situ *hybridization analysis of *Shh *mRNA in whole mounts and on transverse sections of embryos each day between E8.5 and E12.5. We found that at E8.5, *Shh *expression was restricted to a narrow medial domain overlying the prechordal plate extending through the presumptive mammillary and tuberal hypothalamus (Figure 1B-F). At E9.5, the expression in the ventral midline extended anteriorly. However, at the tuberal level *Shh *started to be downregulated medially (Figure 1A1-F1). At E10.5, medial *Shh *expression was expanded into the presumptive preoptic area. In the tuberal hypothalamic primordium, *Shh *was completely excluded from the ventral midline (Figure 1A2-H2). Starting at this stage, Shh expression was also observed in the zona limitans intrathalamica (zli; Figure 1D2). The expression pattern observed at E10.5 persisted over the subsequent days (E11.5 and E12.5), with two changes: the zli was observed on more anterior sections and *Shh *expression was completely absent in the anterior hypothalamic anlage (Figure 1A3-H4). Interestingly, the changes observed in Shh expression are very similar to those described in the developing chick hypothalamus between stages 8 and 22 [8]. Given the dynamic expression pattern of *Shh *in the hypothalamus, the time period when *Shh *is expressed in spatially defined progenitor domains could potentially delineate progenitor populations of distinct hypothalamic nuclei.

### Fate mapping strategy to follow the fate of progenitors in different medial-lateral *Shh-*expressing hypothalamic progenitor domains

**Figure 2:**
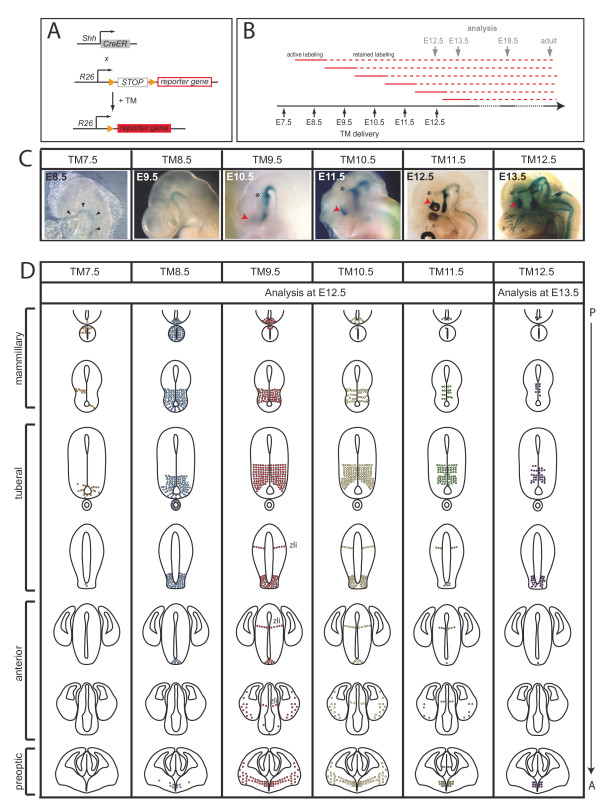
**Changing populations of precursors are marked with Shh-GIFM at different stages of development**. **(A) **Alleles used for GIFM. *Shh*^*CreER *^mice were crossed with *R26 *reporter mice. The reporter gene is either *lacZ *or *EYFP*. Administration of tamoxifen (TM) to pregnant females results in Cre-mediated recombination of the reporter allele and permanent expression of the reporter gene. **(B) **Since TM is only stable for 24 to 36 hours, the period of Cre-mediated recombination is restricted to approximately one day after TM administration (active labeling). Labeling is retained (retained labeling) after Cre activity ceases (due to degradation of TM or downregulation of Cre expression). The retained labeling allows for the fate-mapping of recombined and marked cells. **(C) **X-gal whole mount staining of *Shh*^*CreER/+*^; *R26*^*lz/+ *^embryos one day after the indicated time point of TM administration. (We refer to the time point of TM injection as TM followed by the embryonic day - for example, TM administration at E7.5 is TM7.5, and so on.) Black arrowheads indicate the notochord/prechordal plate, red arrowheads indicate the preoptic area, asterisks indicate the zona limitans intrathalamica (zli). **(D) **Summary of the distribution of *Shh-*expressing cells that were permanently marked at the indicated time points (TM7.5 to TM12.5) and analyzed at E12.5 or E13.5. Cells derived from *Shh-*expressing progenitors are initially found in the ventral midline of the posterior hypothalamic primordium (TM7.5 and TM8.5), but are later (TM9.5 to TM12.5) excluded from the midline. Shh-derived cells are also found in the preoptic area and the zli at later stages, but are almost completely excluded from the anterior hypothalamus.

To investigate whether progenitors in the temporally distinct *Shh*-positive domains give rise to distinct hypothalamic nuclei, we used GIFM [16]. GIFM allows for temporal and spatial control of cell marking by combining an inducible form of site-specific recombinase, CreER with a reporter allele permanently expressing a marker gene (for example, lacZ, enhanced yellow fluorescent protein (EYFP)) upon Cre mediated recombination. Administration of tamoxifen at specific time points in development to activate CreER results in temporal control of cell marking (Figure 2A, B). We used a mouse line in which a TM-inducible form of Cre (CreERT2) is expressed under control of the endogeneous *Shh *promoter and enhancers (*Shh*^*CreER*^) [19,20]. Nuclear translocation of CreER occurs approximately within 6 hours of TM administration and nuclear localization was shown to be maintained for 24 hours [21-23]. Therefore, cells that express *Shh *shortly after TM administration can be genetically marked. For example, administration of TM at E8.5 should result in labeling of cells that express *Shh *between E9.0 and E10.0 [19,20,24]. These cells are then permanently labeled (Figure 2B). To permanently mark *Shh-*expressing progenitors at distinct time points during development and to map the fate of cells derived from the genetically marked progenitors, the ubiquitously expressed *ROSA*^*loxP-STOP-loxP-LacZ *^(*R26*^*lz*^) or *ROSA^loxP-STOP-loxP-EYFP ^*(*R26*^*EYFP*^) alleles were used [25,26]. We refer to the time point of TM injection as TM followed by the embryonic day (for example, TM administration at E8.5 is TM8.5). We showed previously that GIFM results in mosaic marking in the ventral midbrain and we observed mosaic marking also in the hypothalamus (less than 100% of cells expressing *Shh *were marked; compare Figure 1 and Additional files 1 and 2; data not shown). In animals that received TM at the same time point and were analyzed at the same developmental stage, the spatial limits of the domains labeled in this mosaic manner were very similar between animals, but we observed slight variations in the percentage of labeled cells (data not shown).

### *Shh *expression defines distinct precursor domains in the developing hypothalamus

To determine the fate of *Shh-*expressing hypothalamic progenitors, we first assessed whether the progenitor domains that were marked with GIFM corresponded to the dynamic *Shh *expression pattern observed with RNA *in situ *hybridization (Figure 1). Fate-mapped cells derived from progenitors expressing *Shh *at distinct time points (E7.5 to E12.5; Figure 2B) were first analyzed in whole-mount embryos one day after TM administration (Figure 2C). We found that the distribution of the fate-mapped cells at a specific marking time point did indeed correspond to the Shh expression pattern observed at the time of cell marking (Figures 1A1-A3 and 2C; for example, compare TM8.5 with Shh expression at E9.5).

To compare the distribution of the fate-mapped cells between different labeling time points in detail, we also analyzed transverse brain sections at a common embryonic stage (E12.5; or E13.5 for TM12.5; summarized in Figure 2D; Additional files 1 and 2). Shh-GIFM with TM7.5 resulted in the labeling of cells in the notochord and prechordal plate and of a few cells in the ventral midline of the mammillary and tuberal hypothalamic anlage (Figure 2C, D; Additional file 1). Cells derived from *Shh-*expressing progenitors marked at TM8.5 (hereafter referred to as Shh-derived cells) were found throughout the presumptive mammillary and tuberal hypothalamus. In the anterior hypothalamic primordium, cells were labeled at the ventral midline in more posterior sections, while no marking was found in the anterior aspects of the anterior hypothalamus (Figure 2C, D; Additional file 1). A few labeled cells were found in the future preoptic area. With TM9.5, only few marked cells were found in the presumptive mammillary nucleus. Shh-derived cells were excluded from the ventral midline in posterior sections, but were still located medially in the anterior tuberal hypothalamic primordium and the posterior aspects of the anterior hypothalamic primordium. In addition, a few scattered cells were located in the anterior aspects of the presumptive anterior hypothalamus (Figure 2C, D; Additional file 1). Marked cells were also observed in the preoptic area and the zli. With GIFM with TM10.5, TM11.5 and TM12.5, the distribution of the cells was similar to GIFM with TM9.5, but with less cells located in the presumptive mammillary body and anterior hypothalamus (Figure 2C, D; Additional file 2). We also found that less cells were observed in the mantle layer of the developing hypothalamus with TM11.5 and TM12.5. This is most likely due to the fact that the progenitors were marked only shortly before we analyzed the fate of the labeled progenitor cells at E12.5 or E13.5. The time window of less than 24 hours is too short for the generation of differentiated cells from the marked progenitors.

In summary, our analysis shows that our GIFM approach faithfully marks *Shh-*expressing progenitor domains. Based on the distribution of fate mapped Shh-derived cells, we can categorize the changes in cell distribution into the following stages: 'very early' (TM7.5) - only very few Shh-derived cells are found in the posterior hypothalamic primordium; 'early' (TM8.5) - Shh-derived cells are distributed in the mammillary and tuberal regions of the hypothalamic primordium; 'late' (TM9.5 to TM11.5) - Shh-derived cells are in the preoptic area and in the posterior hypothalamic anlage, but are largely excluded from the future mammillary nucleus and the ventral midline of the tuberal hypothalamic primordium; 'very late' (TM12.5) - the distribution of the fate-mapped cells is comparable to TM9.5 to TM11.5, but less cells are labeled (Figure 2C, D).

**Figure 3:**
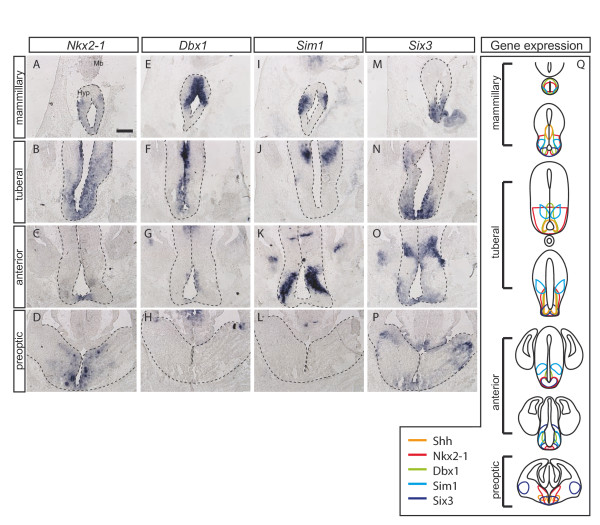
**Transcription factor domains in the developing hypothalamus**. **(A-P) **RNA *in situ *hybridization on E12.5 transverse sections. The ventral diencephalon of selected sections along the anterior-posterior axis is shown. Hyp, hypothalamus; Mb, midbrain. **(Q) **Summary of gene expression domains. Note that only gene expression domains in the hypothalamus are indicated in the schematic, and thalamic gene expression is not indicated. Scale bar: 200 μm.

To investigate whether these distinct domains correlate with the expression of transcription factors known to define specific domains in the hypothalamus, we performed RNA *in situ *hybridization on E12.5 transverse sections of the hypothalamic primordium. *Nkx2-1 *and *Six3 *are expressed in the ventral regions of the hypothalamic primordium (Figure 3A-D, 3M-Q) [27,28]. *Dbx1 *is expressed in the ventricular zone of the posterior hypothalamic anlage, but is excluded from the ventral midline and is not expressed in the preoptic area (Figure 3E-H, 3Q) [29,30]. *Sim1 *is expressed in the progenitors of the anterior periventricular, paraventricular and supraoptic nuclei in the anterior hypothalamus, but expression is also found in the dorsal aspects of the tuberal hypothalamus and in the mammillary region (Figure 3I-L, 3Q) [31]. Comparing the gene expression patterns with the position of the cells derived from *Shh-*expressing precursors marked at different stages of development, we find that cells labeled at the early and very early stage (TM7.5 to TM8.5) are largely restricted to the *Nkx2-1/Six3*-positive domain in the tuberal hypothalamus (Figures 2 and 3A-D, 3M-Q; Additional file 1). In the mammillary region, Shh-GIFM at these early stages marks cells throughout the entire primordium; therefore, they are not restricted to a specific gene expression domain. At late and very late stages of labeling (TM9.5 to TM12.5) the fate-mapped cells are found in the *Dbx1 *expression domain in the tuberal hypothalamus, with the dorsal-most fate-mapped cells in the hypothalamus localized to the *Sim1*-positive domain (Figures 2 and 3I-L, 3Q; Additional files 1 and 2). In the preoptic area, Shh-derived cells are localized to the *Nkx2-1*-expressing zone. Interestingly, all the markers analyzed are also expressed in the anterior hypothalamus, but Shh fate-mapped cells are largely excluded from this area. In summary, hypothalamic progenitors expressing Shh early (before E9.5) or late (after E9.5) are localized to different transcription factor expression domains.

### Two discrete transverse domains of *Shh*-lineage in the hypothalamus

**Figure 4:**
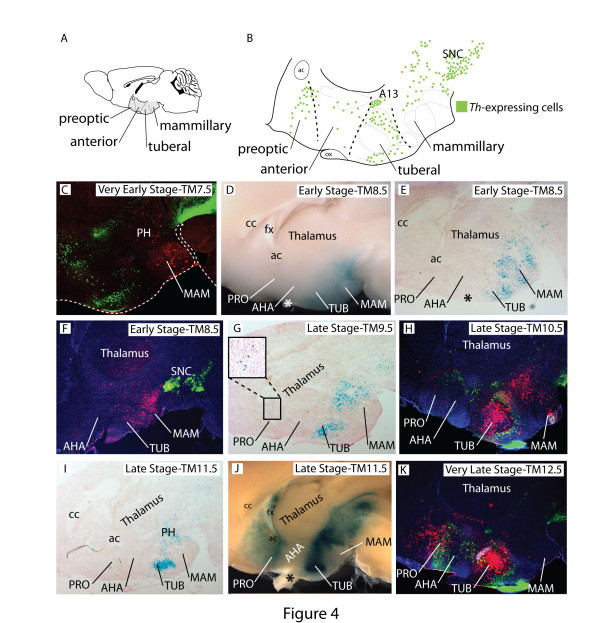
**Two discrete domains of *Shh*-lineage along the rostrocaudal hypothalamic axis**. **(A) **Adult mouse brain in sagittal view showing the hypothalamus (shaded gray) and its four transverse subdivisions. **(B) **Schematic distribution of tyrosine hydroxylase (Th) expressing cells (green dots) on a sagittal view of the hypothalamus (with information from the Allen Brain Atlas [33]). **(C-K) **Brains of *Shh*^*CreER/+*^; *R26*^*lz/+ *^mice. Tamoxifen (TM) was administered as indicated and sections or whole-mount brains were labeled to detect the Shh-lineage. (C, F, H, K) Sagittal sections of adult hypothalamus labeled for Shh lineage (β-gal, red) and Th (green), TM as indicated. Dashed line outlines the brain. (D, J) Right side of hemi-dissected adult brain labeled for Shh-lineage (X-gal reaction, blue), TM as indicated. (E, G, I) Sagittal sections through the E18.5 hypothalamus labeled for Shh lineage (X-gal reaction, blue), TM as indicated. (G) Only few fate-mapped cells are found in the preoptic area with TM9.5. The boxed area is shown in higher magnification. Asterisks indicate the optic chiasm.A13, dopaminergic cell group A13; ac, anterior commissure; AHA, anterior hypothalamic region; cc, corpus callosum; fx, fornix; MAM, mammillary region; Ox, optic chiasm; PH, posterior hypothalamus; PRO, preoptic region; SNC, substantia nigra pars compacta; TUB, tuberal region.

The analysis of Shh-derived cells at E12.5 showed a highly dynamic distribution along the mediolateral as well as the rostrocaudal axis of the developing hypothalamus. To gain insight into how the distinct progenitor domains in the embryonic hypothalamus relate to the four transverse regions in the mature hypothalamus - preoptic, anterior, tuberal and mammillary (Figure 4A) - we first analyzed the distribution of fate-mapped cells in sagittal sections and hemisected brains in the adult (postnatal day 30). To obtain anatomical reference points, we co-stained the sections with an antibody against tyrosine hydroxylase (Th), the rate-limiting enzyme in dopamine synthesis. Th-expressing cells form several distinct groups along the rostrocaudal axis of the ventral diencephalon (Figure 4B; see, for instance, [32] as well as the Allen Brain Atlas expression pattern database [33]). These include numerous periventricular cells in the preoptic region, in the arcuate nucleus and in the dorsomedial nucleus, as well as the so-called A13 group in the zona incerta (Figure 4B). Since the prenatal hypothalamus shows less histological complexity than the adult hypothalamus, but all major nuclei are already recognizable, we analyzed the distribution of fate-mapped cells also in X-gal-labeled sagittal sections at E18.5 (Figure 4E, G, I).

At the very early stage (TM7.5), the few hypothalamic cells derived from *Shh-*expressing progenitors were found in the mammillary body and posterior hypothalamus (Figure 4C). Shh-GIFM at the early stage (TM8.5) labeled cells that were distributed in the caudal hypothalamic domain that included the mammillary region and the ventral part of the tuberal region (Figure 4D-F). Up to this labeling time point, the Shh-derived hypothalamic cells were restricted to one single caudal territory, which originates first with few cells in the mammillary region (very early pattern; Figure 4C) and then expands rostrally to include the ventral tuberal region (early pattern; Figure 4D-F). This change in the distribution of fate-mapped cells in the adult hypothalamus correlates well with the rostral expansion of *Shh-*expressing progenitors from the mammillary recess (TM7.5) to a large, ventral and caudal domain including all ventral midline neuroepithelium of the mammillary and tuberal regions (TM8.5) observed during embryogenesis (Figure 2C, D).

Two striking modifications of this pattern appeared at the late stage (TM9.5, TM10.5 or TM11.5). Rostrally, a novel group of fate-mapped cells appears labeled in the preoptic region (ventral and caudal to the anterior commissure) (Figure 4G-J). Caudally, Shh-derived cells no longer contribute to the ventral part of the mammillary or tuberal region (Figure 4G-J). This distribution again correlates well with the corresponding progenitor domains, which show a decrease in labeling in the mammillary neuroepithelium and the medial tuberal progenitor zone from TM9.5 through TM11.5 as well as a large increase in labeled progenitors in the preoptic region (Figure 2C, D). At this point, a rostral and a caudal domain of Shh-derived cells are visible in the hypothalamus. Since the intervening region (anterior hypothalamic region) is almost devoid of labeled cells, hypothalamic Shh-derived cells from the 'late' domain show a remarkable pattern with alternate labeled and non-labeled bands, which are rostrocaudally arranged (Figure 4J). Finally, cells derived from progenitors expressing Shh at E12.5 (very late stage) showed essentially the same distribution pattern, but the total number of marked cells was decreased (Figure 4K).

In summary, on sagittal sections and hemisected brains a remarkable and consistent heterogeneity in the distribution of Shh-derived cells was apparent. Of the four transverse hypothalamic regions, the most anterior (preoptic) and two most caudal (tuberal and mammillary) showed abundant labeled cells (depending on the time point when *Shh-*expressing progenitors were marked), while the anterior hypothalamic area seemed virtually devoid of them.

### Mediolateral extent of the Shh-lineage hypothalamic domains: most tuberal and mammillary nuclei are of *Shh *descent

**Figure 5:**
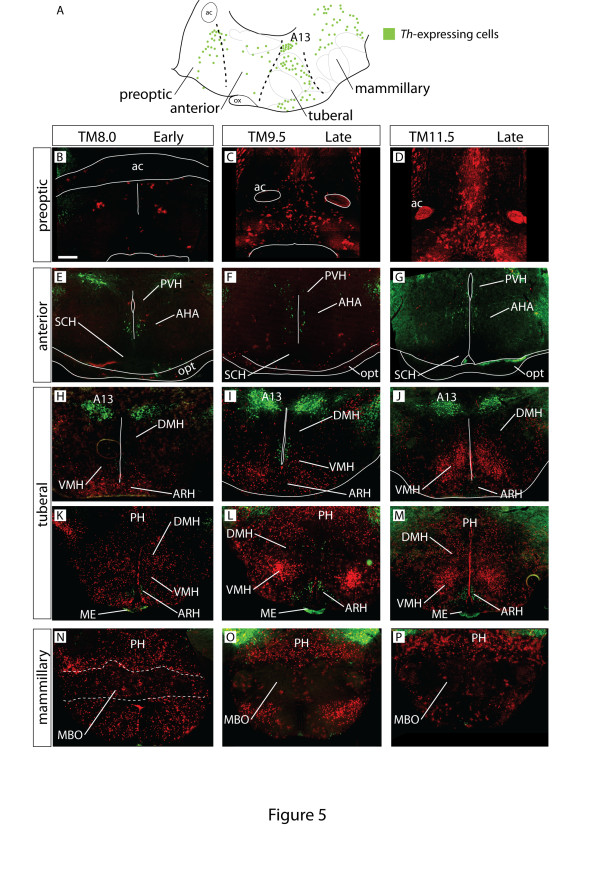
**Preoptic glia and most tuberal and mammillary neurons are of *Shh *descent**. **(A) **Schematic distribution of tyrosine hydroxylase (Th)-expressing cells (green dots) on a sagittal view of the hypothalamus. **(B-P) **Transverse sections of adult mouse brains TM8.0 (B, E, H, K, N), TM9.5 (C, F, I, L, O) and TM11.5 (D, G, J, M, P) labeled for Shh lineage (β-gal, red) and Th (green). (B-D) The preoptic region contains large numbers of late-stage fate-mapped cells with glia-like morphology. (E-G) The anterior hypothalamus contains very few fate-mapped cells at all stages. (H-M) Many fate-mapped cells are located in the tuberal area at all stages, especially in the ventromedial nucleus (VMH). (N-P) Early-stage fate-mapped cells (TM8.5) also contribute strongly to the mammillary body. A13, dopaminergic cell group A13; ac, anterior commissure; AHA, anterior hypothalamic region; ARH, arcuate nucleus; DMH, dorsomedial nucleus; opt, optic tract; MBO, mammillary body; ME, median eminence; Ox, optic chiasm; PH, posterior hypothalamus; PVH, paraventricular hypothalamic nucleus; SCH, suprachiasmatic nucleus; VMH, ventromedial nucleus. Scale bar: 800 μm.

The hypothalamus is subdivided along the mediolateral axis into three zones with distinct histological and functional properties (periventricular, medial and lateral) [3]. To map the mediolateral extent of the Shh-derived hypothalamic territory, we examined X-gal stained transverse sections through the hypothalamus of E18.5 embryos marked at different induction time points (Additional file 3 and data not shown). In addition, to ascertain the precise boundaries of the Shh-lineage domains, we analyzed cells marked with Shh-GIFM in transverse sections of adult brains. These sections were co-stained with Th to obtain anatomical reference points (Figure 5A and see above) and hypothalamic nuclei were identified and named according to reference works [33-36].

#### Early stage

Since Shh-GIFM with TM8.0 or TM8.5 marked cells in the ventral midline of the mammillary and tuberal hypothalamic primordium (Figure 2; Additional file 1; data not shown), we could investigate which hypothalamic nuclei are derived from the medial progenitor zone. Consistent with the analysis of sagittal sections, fate-mapped cells were very rare in the preoptic and anterior hypothalamus, but were widely distributed in the tuberal and mammillary regions (Figure 5B, E, H, K, N; Additional file 3). The most rostral level at which fate-mapped cells could be found was the arcuate nucleus, at the level of rostral end of the A13 *Th*-expressing group (Figure 5H). Caudal to this point, numerous labeled cells were found at all dorsoventral and mediolateral levels of the tuberal and mammillary areas, including the ventromedial and dorsomedial nucleus, the arcuate nucleus, the median eminence (Figure 5K) and the mammillary body (Figure 5N).

#### Late stage

Shh-GIFM with TM9.5 to TM11.5 allowed us to assess which nuclei are derived from lateral progenitor domains in the mammillary and tuberal regions, as well as from the rostral domain (Figure 2C, D). The size and position of the *Shh-*expressing progenitor domains is similar with TM9.5 to TM11.5. Since Shh expression is restricted to the ventricular zone, comparing the distribution of Shh-derived cells labeled with TM9.5, TM10.5 or TM11.5 can help to define the relation between the birth date (the time point when the cells leave the progenitor zone) and the final position of a neuron. With TM9.5, a broad cell population derived from the lateral *Shh-*expressing progenitor domain should be labeled. Based on their spatial origin, the same population could be labeled with TM10.5 and TM11.5. However, since some neurons have already left the progenitor zone at E10.5 or E11.5, the later GIFM time points will label a more restricted cell population, namely of neurons that have a later birth date.

Consistent with the results from the analysis of sagittal sections, the preoptic region showed numerous labeled cells (rostral Shh-lineage domain; Figure 5C, D) whose morphology in most cases suggested glia (see below). Characteristic anterior hypothalamic nuclei like the paraventricular hypothalamic nucleus or the suprachiasmatic nucleus were virtually devoid of Shh-lineage cells, as was the portion of the lateral hypothalamic zone corresponding to this region (Figure 5F, G). Fate-mapped cells contributed extensively to the tuberal region, but were excluded from the ventromedial tuberal hypothalamus, consistent with their origin in a lateral progenitor domain (Figures 2 and 5I, J, L, M; Additional file 3) [8]. The most rostral cells of the Shh lineage in the caudal domain were again found in the arcuate nucleus (Figure 5I). The two large and characteristic nuclei of the tuberal region, the dorsomedial and the ventromedial nuclei, showed interesting differences in contribution of fate-mapped cells. The ventromedial nucleus (tuberal region) appeared as a large and compact group of Shh-derived cells packed together and showing a clearly recognizable histological pattern (Figure 5I, J, L, M; Additional file 3). In contrast, the dorsomedial nucleus contained Shh-derived cells, which do not seem to aggregate into a histologically recognizable nucleus (Figure 5I, J, L, M). Interestingly, fate-mapped cells were also found in the lateral aspects of the tuberal hypothalamus with GIFM between TM9.5 and TM11.5, indicating that cells that leave the progenitor zone after E10.5 or E11.5 (have a later birth date) can still contribute to the lateral hypothalamus. Finally, very few cells were marked in the mammillary nucleus with Shh-GIFM with TM10.5 and TM11.5 (Figure 5P, data not shown).

#### Very late stage

Shh-GIFM with TM12.5 labeled numerous cells in the lateral hypothalamus of the tuberal region, in the preoptic region and in the ventromedial nucleus, which was clearly delimited (data not shown). Many of these cells (virtually all in the preoptic region) showed astrocytic morphology (see below). Although the general pattern at this stage was similar as in the previous, the total number of cells was decreased, and cells with glial morphology were most numerous.

In summary, the analysis of sagittal (Figure 4) and transverse (Figure 5; Additional file 3) brain sections demonstrates clearly that the anterior hypothalamic region is not derived from *Shh-*expressing cells. This set of data suggests that there is little or no cell mixing between the tuberal and anterior or the preoptic and anterior hypothalamus. In other words, the cells produced by a certain progenitor domain tend to remain in the same rostrocaudal level.

### Late born hypothalamic neurons settle in medial and lateral aspects of the tuberal hypothalamus

**Figure 6:**
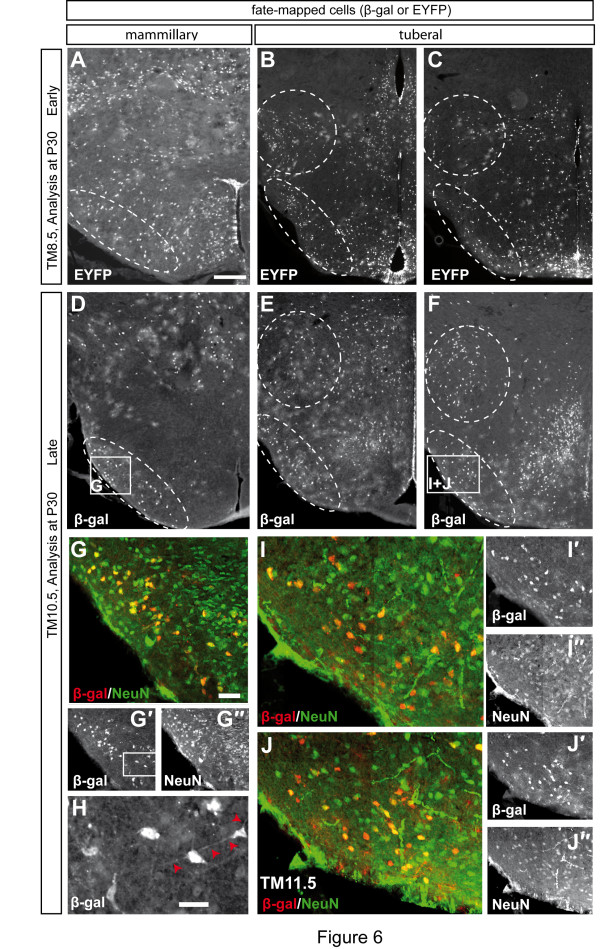
**Hypothalamic neurons derived from progenitors that express Shh after E10.5 settle in medial and lateral aspects of the tuberal hypothalamus**. **(A-F) **Early and late stage fate-mapped cells contribute to the lateral tuberal and mammillary hypothalamus. Ventro- and dorsolateral areas of the hypothalamus are circled with a dashed line. **(G-I) **Immunostaining for fate-mapped cells (β-gal, red) and NeuN, a pan-neuronal marker (green). Pictures were taken using a Zeiss Apotome set-up. Late-stage fate-mapped cells overlap with the neuronal marker NeuN and have neuronal morphology (H). Red arrowheads indicate neuronal processes. Scale bar: 800 μm (A-F); 100 μm (G, I, J); 20 μm (H).

Based on birth dating studies, it has been proposed that the subdivision of the hypothalamus into three mediolateral zones (periventricular, medial and lateral) is due to an 'outside-in' generation of neuronal layers and clusters, in which the earliest born neurons migrate to lateral positions, while later born neurons stay close to the hypothalamic ventricular zone [37]. Interestingly, we found that fate-mapped cells that are derived from progenitors that express Shh after E10.5 or even after E11.5, and should therefore be 'late' born cells, settle in all mediolateral aspects of the tuberal hypothalamus. To investigate whether these cells are neurons, we performed double immunostainings for fate-mapped cells and the neuronal maker NeuN. Indeed, cells in the lateral hypothalamus that are derived from *Shh-*expressing cells marked with TM9.5, TM10.5 or TM11.5 overlap with NeuN and also display a neuronal morphology (Figure 6D-J and data not shown). *Shh-*expressing cells that were labeled with TM8.5 also contributed neurons to these lateral regions of the tuberal hypothalamus (Figure 6A-C and data not shown). These data indicate that at least a subset of lateral hypothalamic cells are derived from 'late' born neurons that likely migrate from the ventricular zone through pre-existing differentiated neuronal layers to reach their final position. These results suggest that the proposed three mediolateral waves of hypothalamic neurogenesis orderly apposing each other from 'outside-in' [17] might not be a general mechanism underlying the generation of the different mediolateral zones.

### *Shh-*expressing progenitors give rise to astrocytes in the hypothalamus

**Figure 7:**
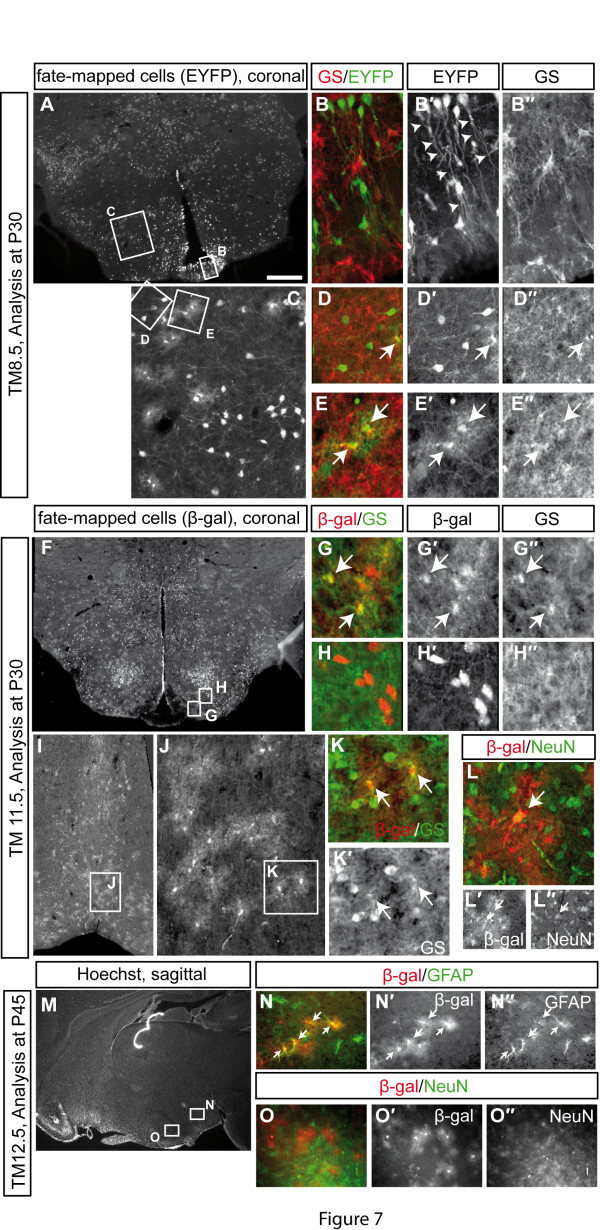
***Shh-*expressing progenitors give rise to astrocytes in the hypothalamus**. Immunostaining for glutamin synthetase (GS) and fate-mapped cells (β-gal or EYFP) (B, D, E, G, H, K), GFAP and β-gal (N) or NeuN and β-gal (L, O). Sections were imaged using a Zeiss Apotome setup. **(A, B) **In the tuberal hypothalamus, fate-mapped cells (TM8.5, green) are localized to the median eminence and display the typical morphology of tanycytes (arrowheads). **(C-H) **Tuberal hypothalamus. Fate-mapped cells with astrocytic morphology overlap with GS (arrows), and marked cells with neuronal morphology do not express GS. **(I-K) **Almost all fate-mapped cells in the preoptic area have astrocytic morphology and overlap with GS. **(L) **Only few fate-mapped cells in the preoptic area overlap with the neuronal marker NeuN. **(M-O) **Progenitors expressing Shh after E12.5 give rise to astrocytes but not neurons. Arrows indicate double-labeled cells. Scale bars: 800 μm (A, F, I); 100 μm (C, J, N, O); 40 μm (B, D, E, G, H, K, L). P, postnatal day.

While analyzing the distribution of fate-mapped cells in adult brain sections we noticed that at all labeling stages (Shh-GIFM at E7.5 to E11.5), fate-mapped cells showed two clearly distinct morphologies. In addition to neurons with large, strongly labeled cell bodies and axonal projections and/or dendrites, cells with smaller cell bodies surrounded by small and highly branched processes were detected throughout the hypothalamus (Figures 5 and 6 and data not shown). Since the latter morphology is indicative of astrocytes, we performed immunostainings for β-gal and glutamine synthetase, an established broad astrocyte marker [38,39] or for β-gal and the astrocyte marker glial fibrillary acidic protein (GFAP) [40]. We found that β-galactosidase (β-gal)-positive cells with an astrocytic morphology indeed expressed glutamine synthetase and GFAP (Figure 7). Interestingly, the vast majority of fate-mapped cells in the preoptic area (marked with Shh-GIFM from E9.5 to E12.5) displayed this typical astrocytic morphology, while only few preoptic neurons appeared to be derived from *Shh-*expressing progenitors (Figures 5B-D and 7I-L and data not shown). With TM12.5, a majority of labeled cells were astrocytes, likely because at this time point neurogenesis starts to cease in the hypothalamus and progenitors give rise to glial cell types instead (Figure 7M-O). Shh-expressing cells fate-mapped with TM8.5, but not at any other marking time point, appeared to give rise to tanycytes in the median eminence, a specialized type of glia cell that is in contact with the pituitary [41]. Marked cells that were localized to the median eminence displayed the typical tanycyte morphology (Figure 7A, B and data not shown). Since only progenitors fate-mapped with TM8.5 are found in the ventral midline of the tuberal hypothalamic primordium, these results strongly suggest that tanycytes are derived from progenitors in the ventral midline.

## Discussion

**Figure 8:**
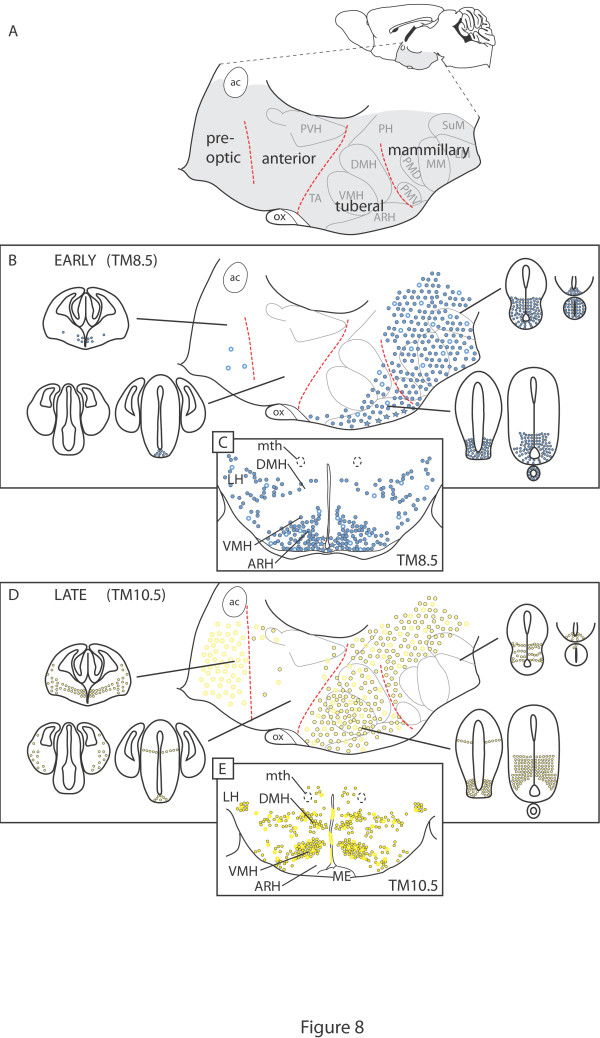
**Distribution of Shh-lineage cells from the different progenitor domains in the mouse adult hypothalamus**. **(A) **Adult mouse brain in sagittal view showing the hypothalamus (shaded gray). **(B, D) **Summary of distribution of cells fate-mapped at early and late stages. Magnified view of the shaded area showing the transverse hypothalamic subdivisions and some of the largest nuclei and Shh-lineage progenitor domains in the E12.5 neuroepithelium (small profiles; see also Figure 2). In the schematic of the adult hypothalamus, closed circles indicate neurons, open circles indicate astrocytes, and stars indicate tanycytes. **(C, E) **Transverse section through the adult tuberal hypothalamus. ac, anterior commissure; ARH, arcuate nucleus; DMH, dorsomedial nucleus; LH, lateral hypothalamus; LM, lateral mammillary nucleus; ME, median eminence; MM, medial portion of mammillary body; mth, mammillothalamic tract; ox, optic chiasm; PH, posterior hypothalamus; PMD, dorsal premammillary nucleus; PMV, ventral premammillary nucleus; PVH, paraventricular hypothalamic nucleus; SuM, supramammillary nucleus; TA, tuberal area; VMH, ventromedial nucleus.

Far from being a specialized subject, hypothalamic regionalization offers insight into questions of general importance in neural development. We have applied for the first time Shh-GIFM to this famously complex structure and our results shed light on the nature and organization of Shh progenitor domains in the neural tube, neuronal migration, patterns of neurogenesis and the origin of hypothalamic complexity. The results are summarized in Figure 8.

### Ventral diencephalic and ventral mesencephalic domains

Early in development, the *Shh *expression domain in the ventral neural tube starts rostrally in the ventral rostral diencephalon (characterized here as the progenitor domain of the tuberal and mammillary regions) and extends caudally uninterrupted through the ventral midbrain, hindbrain and spinal cord. Comparison of our data with recent analyses of Shh-derived cells in the ventral midbrain [19,42,43] indicates that the domains we observe in the caudal hypothalamus are indeed continuous (although generated earlier) with the *Shh-*expressing progenitor domains of the ventral midbrain, suggesting a continuity of rostrocaudal, ventral regions along the neural tube, modulated by local signals. The *Shh-*expressing progenitor domain appearing in the preoptic region later in development is, however, not part of this continuous domain.

### *Shh*-expressing progenitor domains and restricted gene expression

Progenitor domains are characterized by complex patterns of transcription factor gene expression, and an important yet unresolved question concerns the molecular determinants of the neurons produced in each progenitor domain. An initial mapping of progenitor domains in the hypothalamus and the neuronal nuclei they generate has been made based on the fact that some transcription factors are expressed not only in restricted progenitor domains but also in the neurons they generate [12] and similar approaches are contributing to unravel the relation between progenitor domain and mantle layer in the hypothalamus [44]. Through recent genomic studies, a glut in resources has been made available that will be key to answer this question [45]. The present study shows that the large Shh-expressing domains are formed by smaller, partially overlapping domains. This corresponds with the ample variety of Shh-derived hypothalamic neuronal nuclei. Intriguingly, the early- and late-expressing domains show differential expression of determinants, pointing at the molecular basis of neurogenetic timing.

### Multiple, differential patterns of neurogenesis in the Shh progenitor domains

The neuroepithelial progenitor domains are characterized by restricted gene expression patterns [12,46] and generate specific neurons according to distinctive rhythms [47]. Some specific hypothalamic neuronal groups are formed in a single neurogenetic peak [48] while others have distinct subgroups generated at different times [49,50]. Are *Shh-*expressing hypothalamic precursors characterized by a specific timing and rate of neurogenesis? The answer is negative. Our data show that the hypothalamic Shh-domains generate many neurons for many nuclei through differential neurogenetic dynamics. As an example, the lateral hypothalamus (in the tuberal and mammillary regions) is generated as a large number of scattered cells over an extended period of time and an extended area of the brain. The two major tuberal nuclei, the ventromedial and the dorsomedial nucleus, generated by opposing strategies, are the best example. While the dorsomedial nucleus seems to receive a steady contribution of scattered cells by the neuroepithelium during TM8.0 through TM12.5 (the same strategy applies to the lateral hypothalamus), the ventromedial nucleus receives massive amounts of neurons, which aggregate forming a histologically recognizable nucleus. In conclusion, the hypothalamic *Shh-*expressing progenitors do not share common neurogenetic dynamics, suggesting that they respond to proliferation cues differentially, according to specific programs and critical periods.

### Hypothalamic patterns of neurogenesis: three waves?

The above paragraph has broader implications. Classically, it has been described that the hypothalamus is formed in three neurogenetic waves producing neurons that accumulate progressively more medial (outside-in) [17]. This has been considered as fundamentally proven, although opposite findings relative to specific hypothalamic neuronal subgroups have been reported [48,51,52]. Our data show that since the *Shh-*expressing progenitor domains remain essentially unchanged (that is, they do not shrink or expand) after E9.5, GIFM with TM9.5, TM10.5 and TM11.5 labels essentially the same progenitor domains, which are generating neurons fated for the same regions. Therefore, by comparing these three stages we can actually make a statement about which cells are born early (before E11.5) or late (after E11.5). If there were distinct waves of neurogenesis generating progressively more medial neuronal zones, they would be obvious in our material (Figures 5 and 6), but they are not.

In conclusion, for a period of several days, the hypothalamic *Shh-*expressing progenitors generate neurons fated for every mediolateral level, and do not generate neurons for a specific mediolateral level at a specific time. At least for these progenitors, the three wave model [17] has to be revised.

### *Shh*-derived cells remain within the same region of the progenitor domain

Our results contribute to clarify the controversy about the modes of hypothalamic migration. Analyses of hypothalamic neurogenesis with tritiated thymidine suggested a pattern of migration in the mediolateral plane ('radial' migration) [17]. Clonal analyses of the avian ventral forebrain [53] and the distribution of specific hypothalamic neurons at different developmental stages in the rat [52] provided confirmatory evidence, although other diencephalic areas show some widely dispersed clones [54,55]. In addition, intrahypothalamic tangential migration as well as migration from the olfactory placode, telencephalon and diencephalon into the hypothalamus have been described [44,56-58]. Here we show that the progenitor domains coincide with the regions where the Shh-derived cells settle, suggesting little or no mixing of cells belonging to different domains. This is clearly confirmed by the fact that neither the preoptic nor the anterior regions are colonized by Shh-derived neurons from caudal regions. Therefore, the Shh-derived hypothalamic cells, that is, most of the tuberal and mammillary regions, migrate mostly in the mediolateral plane (radially) and not in the rostrocaudal plane (tangentially) (with the caveat that short tangential migrations of Shh-derived cells inside the Shh-derived domain would not be detected by our approach).

### The retrochiasmatic area

The retrochiasmatic area is a small ventral and medial hypothalamic region between the optic chiasm (rostrally) and the arcuate nucleus (caudally). On the basis of Shh expression [18,59], this region represents the very rostral tip of the basal plate of the hypothalamus. Therefore, the corresponding neuroepithelium represents also the rostral end of the *Shh-*expressing progenitor domain (Figure 2), and the retrochiasmatic area is labeled in our material (Additional file 3A). Classically classified as part of the anterior hypothalamic region [3], this area is anatomically continuous with and functionally related to the arcuate and ventromedial nuclei of the tuberal region [60-62]. We have represented it (Figure 2) as part of the anterior region as is traditional, but this does not modify our conclusion that the anterior hypothalamus is not Shh-derived.

### The non-Shh-derived hypothalamus: open questions

The embryonic preoptic region has been considered telencephalic as opposed to hypothalamic based on morphological criteria as well as gene expression patterns [59,63]. Moreover, since Shh is a key ventral determinant in the neural tube, it follows that in the E12.5 embryo the caudal, *Shh-*expressing half of the hypothalamus is actually ventral, and the rostral half shares the dorsal portion with the telencephalon [18,59]. Using GIFM, we demonstrate that Shh progenitor domains of the caudal half of the hypothalamus do not produce cells for the preoptic or anterior regions in the adult hypothalamus. In this sense, the two 'rostral' regions of the adult hypothalamus, preoptic and anterior, derive from the dorsal portion of the neural tube, together with the telencephalon. Obviously, the simple longitudinal versus transverse subdivision that works so well in the spinal cord breaks down in the hypothalamus and telencephalon; therefore, it might be time to redefine a novel dorsal subdivision ventral to the telencephalon. Furthermore, it remains to be investigated which hypothalamic neurons originate in the telencephalon [56,64,65] and whether they preserve any anatomical or functional links with their region of origin.

### From complex specification to complex adult morphology

The adult hypothalamus is functionally organized into a behavioral control column [2,62] whose rostrocaudal arrangement obscures the developmental seams between the regions derived from specific progenitor domains as revealed by GIFM. Even so, the developmental heterogeneity we unveil here probably contributes to the staggering functional complexity of the hypothalamus. This is perhaps particularly true of the preoptic and anterior regions, which are the most complex hypothalamic regions and, as has been observed, the ones causing the most confusion [3,56,66]. Likewise, finding out the origin of that portion of the lateral hypothalamus corresponding to the non-Shh-derived regions promises to be of great interest.

## Conclusions

The *Shh*-expressing progenitor domains in hypothalamus are continuous (although generated earlier) with the *Shh-*expressing progenitor domains of the ventral midbrain, and can be seen as the most rostral part of the longitudinal, ventral progenitor regions continuous along the neural tube.

The behavioral control column that constitutes the adult hypothalamus has its origin in a variety of progenitor domains in the ventral midline and in the basal and alar portions of the rostral diencephalon. Of its four transverse subdivisions, the two most caudal (mammillary and tuberal regions, including the retrochiasmatic area) are formed mostly by neurons and glia originated in *Shh-*expressing progenitor domains of the ventral midline and basal plate. The anterior hypothalamic region is virtually devoid of neurons produced in said domains. The most rostral hypothalamic region (preoptic region) receives mostly glia from an independent, rostral and late-appearing *Shh-*expressing domain.

There is almost no tangential migration from *Shh*-derived regions into the non-*Shh*-derived anterior hypothalamic region. This suggests the existence of molecular mechanisms preventing the mixing of cells generated in the basal and alar plates of the rostral diencephalon.

The hypothalamic *Shh-*expressing domains are formed by smaller, partially overlapping domains of transcription factor gene expression. The hypothalamic progenitors expressing *Shh *early are localized to transcription factor expression domains (*Nkx2-1*/*Six3*) different from those of progenitors expressing *Shh *late (*Dbx1*/*Sim1*).

The hypothalamic *Shh-*expressing progenitor domains generate neurons fated for all mediolateral levels during a protracted neurogenetic period of several days. No marked outside-in waves of neurogenesis could be detected in our material.

## Materials and methods

### Fate mapping

Animal studies were approved by the University of Bonn Animal Care and Use Committee. *ROSA*^*loxP-STOP-loxP-LacZ *^(*R26*^*lz*^) reporter mice were kindly provided by Dr P Soriano [25]; *ROSA*^*loxP-STOP-loxP-EYFP *^(*R26*^*EYFP*^) were kindly provided by Dr F Costantini [26]; *Shh*^*CreER *^were kindly provided by Dr C Tabin [19,20]. Mice heterozygous for the *R26^lz ^*or *R26^EYFP ^*allele and the *CreER *alleles were genotyped as previously described [25,67]. All mice were maintained in an outbred SW or CD1 background. *Shh*^*CreER/+ *^; *R26*^*lz/lz *^or *Shh*^*CreER/+ *^; *R26*^*EYFP/EYFP *^males were bred with SW or CD1 wild-type females (Taconic, Hudson, NY, USA or Charles River, Wilmington, MA, USA) to generate *Shh*^*CreER/+*^; *R26*^*lz/+ *^or *Shh*^*CreER/+*^; *R26*^*EYFP/+ *^progeny. Noon of the day that a vaginal plug was detected was designated as E0.5. TM (Sigma T-5648, St. Louis, Mo, USA) was dissolved in corn oil (Sigma C-8267) at a final concentration of 20 mg/ml. Pregnant females were given 3 to 4 mg TM through oral gavage with animal feeding needles (Fisher Scientific, Waltham, Ma, USA or Fine Science Tools, Heidelberg, Germany) at 12 pm each day between E7.5 and E12.5 and at midnight of E7.5 (corresponding to E8.0). For the analysis of fate-mapped cells at postnatal stages, the TM solution contained progesterone (Sigma P-0130) at a concentration of 5 mg/ml to reduce the incidence of miscarriages.

### Tissue processing, RNA *in situ *hybridization and immunofluorescent stainings

Embryos or embryonic brains were dissected and fixed in 4% paraformaldehyde for 20 to 90 minutes. Postnatal mice were perfused intercardiacally with 4% paraformaldehyde, and brains were dissected and postfixed in 4% paraformaldehyde overnight. E8.5 to E13.5 embryos or embryonic brains were sectioned on a cryostat at 12 μm, E18.5 brains at 14 μm and postnatal brains at 40 μm (free-floating sections). For RNA *in situ *hybridization, frozen sections were used or the tissue was manually processed for paraffin embedding and sectioned at 7 μm. RNA *in situ *hybridization was performed as described [19]. RNA *in situ *probes were obtained from AL Joyner (Shh, Nkx2.1), JL Michaud (Sim1), K Campell (Dbx1) and G Oliver (Six3).

X-gal and immunofluorescent staining on sections was performed using standard procedures [19]. Primary antibodies were: goat anti-β-gal (1:2,000; Biogenesis Kingston, NH, USA), rabbit or rat anti-green fluorescent protein (1:400, Invitrogen, Carlsbad, CA, USA or 1:2,000, Nacalai, Kyoto Japan) rabbit or mouse anti-tyrosine hydroxylase (1:500; Millipore, Billerica, MA, USA), rabbit anti-calbindin (1:5,000; Swant, Marly, Switzerland), rabbit anti-GFAP (1:500; Millipore), mouse anti-glutamine synthetase (1:500; Millipore). Secondary antibodies were: donkey anti-goat IgG-Alexa 555 and donkey anti-rabbit IgG-Alexa 488 (1:500; Invitrogen); donkey anti-goat Cy3, donkey anti-rabbit Cy3 or FITC, donkey anti-mouse Cy3 or FITC (1:200; Jackson ImmunoResearch, West Grove, PA, USA).

### X-gal whole mount stainings

X-gal staining was performed as described [68]. Embryos older than E10.5 were cleared in benzyl-benzoate.

## Abbreviations

β-gal: β-galactosidase; E: embryonic day; EYFP: enhanced yellow fluorescent protein; GFAP: glial fibrillary acidic protein; GIFM: genetic inducible fate mapping; Shh: Sonic hedgehog; TM: tamoxifen; zli: zona limitans intrathalamica.

## Competing interests

The authors declare that they have no competing interests.

## Authors' contributions

SB designed the experiments; SB and FAP performed the experiments; SB and GAB analyzed the data and wrote the manuscript. All authors read and approved the final manuscript.

## Supplementary Material

Additional file 1 **Labeling *Shh-*expressing hypothalamic progenitor domains, TM7.5 to TM9.5**. Series of transverse sections at low (left) and high (right) magnification through the E12.5 diencephalon showing the original lineage labeling at TM as indicated. Click here for file

Additional file 2 **Labeling *Shh-*expressing hypothalamic progenitor domains, TM10.5 to TM12.5**. Series of transverse sections at low (left) and high (right) magnification through the E12.5 diencephalon (E13.5 in the case of TM12.5) showing the original lineage labeling at TM as indicated. Click here for file

Additional file 3 **Hypothalamic Shh-derived lineage in transverse sections**. Two series of transverse sections through the mouse caudal hypothalamus at E18.5 labeled by the X-gal reaction after Shh-GIFM (TM as indicated). AHA, anterior hypothalamic region; ARH, arcuate nucleus; DMH, dorsomedial nucleus; fr, **retroflex fascicle**; LH, lateral hypothalamus; MBO, mammillary body; ME, median eminence; mth, mammillothalamic tract; PH, posterior hypothalamus; RCh, retrochiasmatic nucleus; SUM, supramammillary nucleus; VMH, ventromedial nucleus. Click here for file
