# Supplementary figures and images for: Sonic hedgehog lineage in the mouse hypothalamus: from progenitor domains to hypothalamic regions
Source: Neural Dev. 2012 Jan 20;7:4. doi: 10.1186/1749-8104-7-4 (PMC3292819; doi:10.1186/1749-8104-7-4)

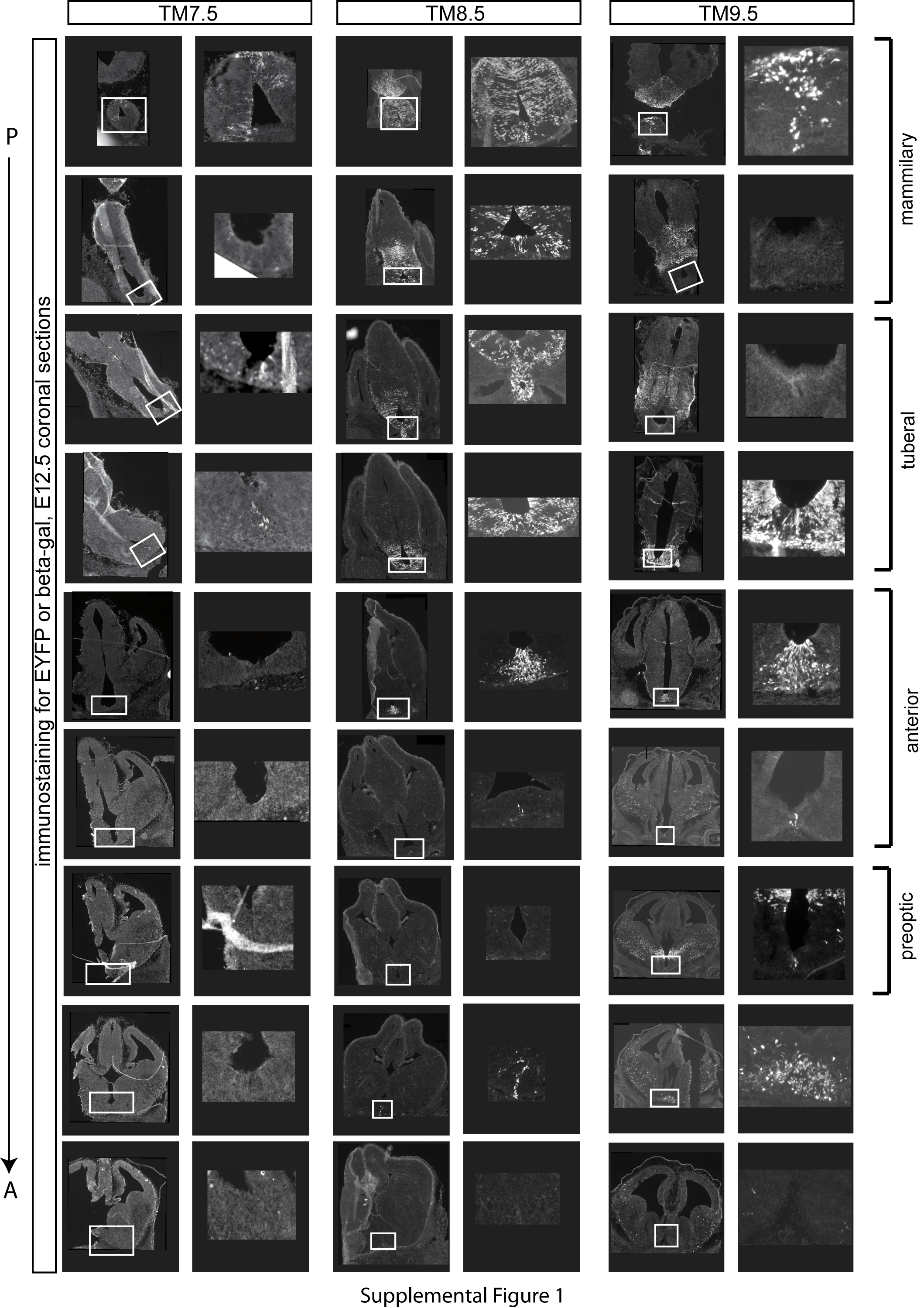

Supplement: Additional file 1 — Labeling Shh-expressing hypothalamic progenitor domains, TM7.5 to TM9.5. Series of transverse sections at low (left) and high (right) magnification through the E12.5 diencephalon showing the original lineage labeling at TM as indicated. [file 1749-8104-7-4-S1.PNG]

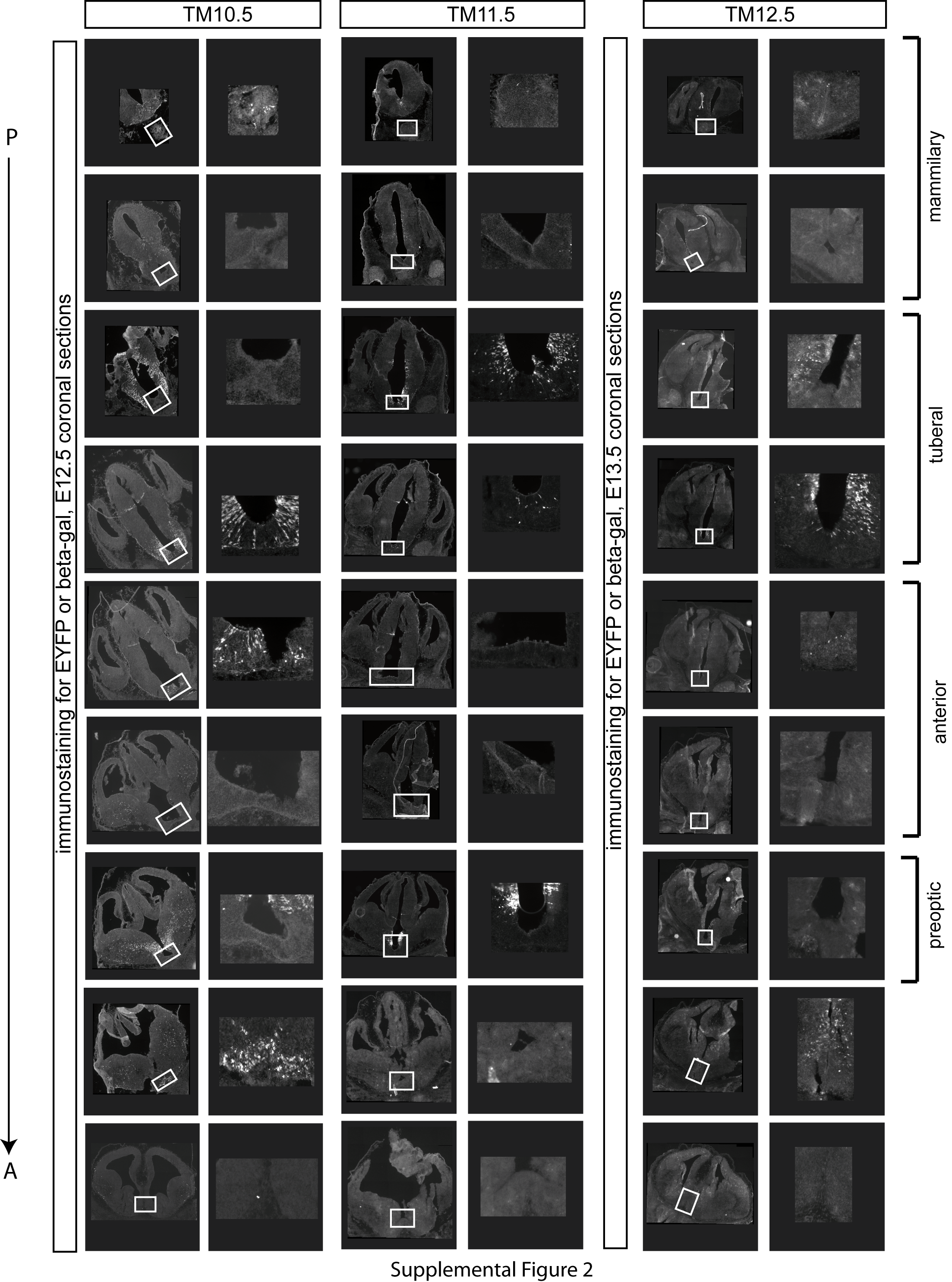

Supplement: Additional file 2 — Labeling Shh-expressing hypothalamic progenitor domains, TM10.5 to TM12.5. Series of transverse sections at low (left) and high (right) magnification through the E12.5 diencephalon (E13.5 in the case of TM12.5) showing the original lineage labeling at TM as indicated. [file 1749-8104-7-4-S2.PNG]

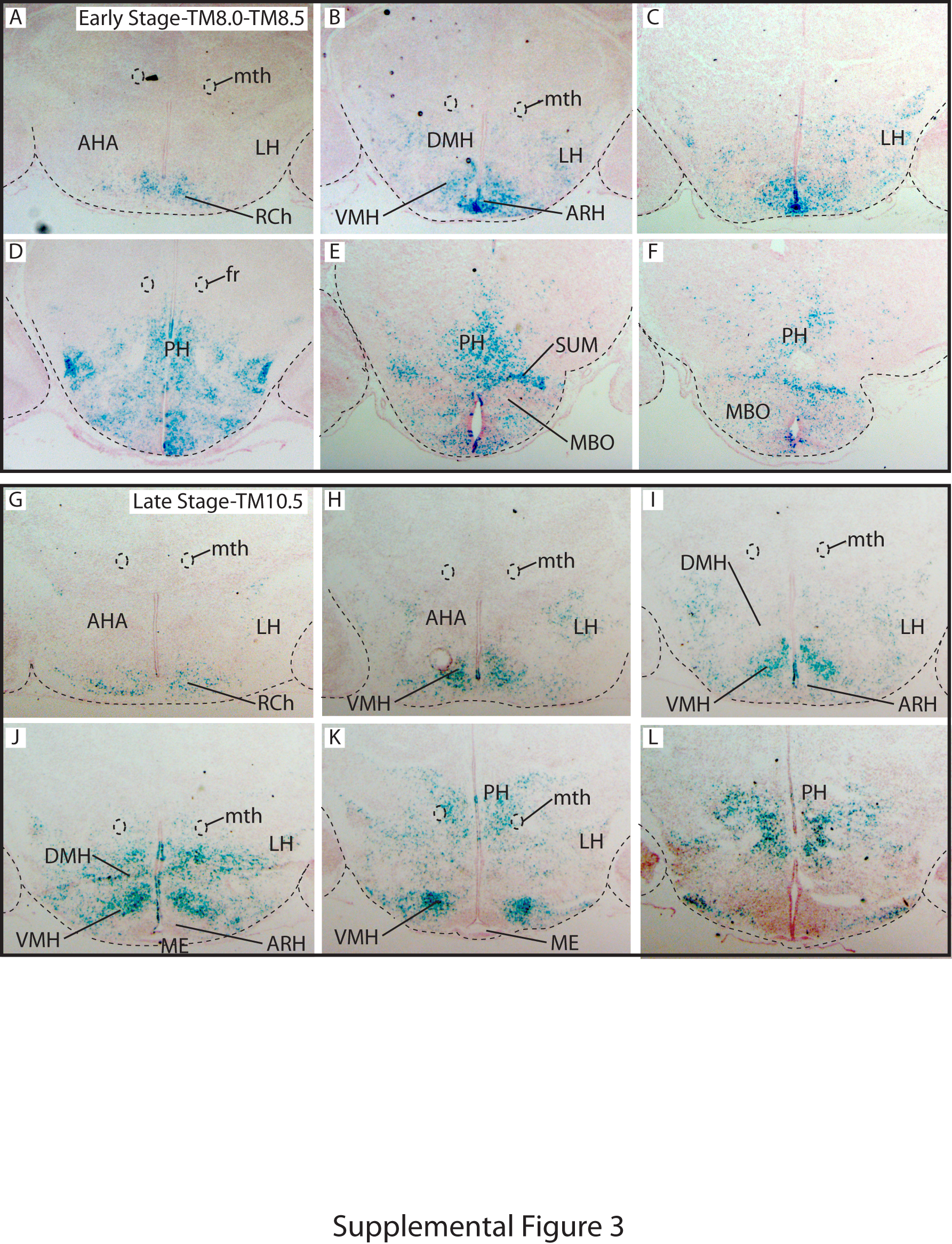

Supplement: Additional file 3 — Hypothalamic Shh-derived lineage in transverse sections. Two series of transverse sections through the mouse caudal hypothalamus at E18.5 labeled by the X-gal reaction after Shh-GIFM (TM as indicated). AHA, anterior hypothalamic region; ARH, arcuate nucleus; DMH, dorsomedial nucleus; fr, retroflex fascicle; LH, lateral hypothalamus; MBO, mammillary body; ME, median eminence; mth, mammillothalamic tract; PH, posterior hypothalamus; RCh, retrochiasmatic nucleus; SUM, supramammillary nucleus; VMH, ventromedial nucleus. [file 1749-8104-7-4-S3.PNG]
